# A Tale of Two Countries: DiaSorin Molecular’s Rapid Response to the COVID-19 Pandemic

**DOI:** 10.3389/fcimb.2022.840210

**Published:** 2022-04-21

**Authors:** Michelle M. Tabb, Giulia Minnucci, Vincenzo Albano

**Affiliations:** ^1^ DiaSorin Molecular LLC, Cypress, CA, United States; ^2^ DiaSorin S.p.A., Gerenzano, Italy

**Keywords:** COVID-19, SARS-CoV-2, Simplexa, pandemic, DiaSorin Molecular, LIAISON MDX, diagnostics manufacturer

## Abstract

In the summer of 2019, DiaSorin Molecular started designing a multiplex respiratory panel with pan-coronavirus detection as one of the planned targets. The R&D team in Gerenzano, Italy was already searching databases, performing alignments and assessing preliminary target regions for common coronavirus RT-PCR, including SARS and MERS-CoV. In December 2019, we were vigilant and following a cluster of pneumonia cases with undetermined etiology in Wuhan, China. As we now know, the cause of the respiratory infections was the new SARS-CoV-2 virus. DiaSorin Molecular swiftly responded in line with our heritage and company history in detecting emerging infectious diseases. Early in the pandemic and in record time, using research and development teams in both Italy and the U.S. together with the U.S. manufacturing team, we were able to develop and commercialize a new diagnostic test, Simplexa™ COVID-19 Direct, to detect SARS-CoV-2. Our unique platform allowed development of a rapid diagnostic test without the need for extraction reagents. Challenges with control materials, quarantines, clinical samples, raw materials and production were overcome and the entire company worked side by side for accelerated delivery of this assay to clinical labs in Europe, the U.S. and Canada.

## Early Development January – February 2020

The first viral genomic sequences of SARS-CoV-2 were available in public databases in mid-January 2020. At the time, it was unclear if this new virus would be a limited outbreak, an epidemic, or something more. In the “business of clinical testing,” there must be a clinical need together with a market and business case to justify commercializing a new assay, and it was challenging to dedicate resources to design an assay exclusively dedicated to a novel virus. To be prepared, the Italian R&D team pivoted from a pan-coronavirus design for a multiplex panel to a stand-alone SARS-CoV-2 assay. The design chosen employed a unique approach using a two-target algorithm that would be more robust and resistant to potential mutations in the new virus. The assay targeted the viral S and ORF1ab genes and utilized fluorescently labeled probes together with corresponding forward and reverse primers to simultaneously amplify both targets. The S gene encodes the spike glycoprotein of the virus and was targeted to specifically detect the presence of SARS-CoV-2 while the ORF1ab region encodes well-conserved non-structural proteins and therefore is less susceptible to recombination. The design had taken advantage of the existing Simplexa™ Direct chemistry formulated for detection of other RNA viruses like influenza and enterovirus, together with LIAISON^®^ MDX instrument parameters. The SARS-CoV-2 primers and probes were combined with the core Simplexa™ Direct chemistry which enables direct detection of pathogens without the need for a separate nucleic acid extraction step or extraction reagents. The chemistry utilizes enzymes and a special buffer system that are resistant to inhibitors commonly found in clinical specimens. The innovative and now patented design was a completely different approach than those developed by the Centers for Disease Control and Prevention (CDC) or World Health Organization (WHO). On January 22, 2020, orders were placed for the first candidate primers and probes for the new assay. However, there was still a lot of uncertainty surrounding the spread of the virus. Then, we all waited like the rest of the world, and watched as the case count started to climb.

On January 30, 2020, the WHO declared the COVID-19 outbreak a Public Health Emergency of International Concern and on the same day, DiaSorin Molecular became aware that the FDA was making an Emergency Use Authorization (EUA) template for test developers to follow. The following day, the U.S. Department of Health and Human Services Secretary declared a Public Health Emergency for the U.S. Subsequently, on February 4, 2020, the Department of Health and Human Services issued a Declaration under the Public Readiness and Emergency Preparedness Act for Medical Countermeasures Against COVID-19, which allowed the FDA to issue EUAs beginning with the CDC’s 2019 Novel Coronavirus Real-Time RT-PCR Diagnostic Panel.

With the EUA path opened, DiaSorin Molecular accelerated assay development. On Saturday, February 7, 2020, at risk of impacting other development activities in favor of a virus with an uncertain future, the first pilot production lot was built.

## Primer, Probe, and Positive Control Challenges

Before the pandemic, DiaSorin Molecular had two qualified oligonucleotide vendors that supplied primers and probes. When it was apparent that the best means for detecting SARS-CoV-2 was using molecular diagnostics, commercial manufacturers, hospitals, and reference labs began ordering from the main oligonucleotide suppliers, which were quickly overwhelmed with demand. In addition, our primary oligonucleotide supplier was awarded a government contract to produce CDC SARS-CoV-2 assay components, and shifted their manufacturing and attention accordingly. We switched to several smaller suppliers to circumvent this challenge and validated a slightly lower purity scale that could deliver equivalent performance. While this had the benefit of actual delivery of material to finish development and produce kits, many smaller oligonucleotide lots were constantly received from multiple vendors, requiring a high level of attention for supply chain management, incoming testing and inventory management.

For typical assay development projects, there are commercial sources or repositories of characterized viral strains for testing, benchmarking assay performance, and kit positive controls. One of the first development challenges encountered with this new virus was obtaining characterized reference material for testing. Initially, there were no sources of virus other than those obtained from patient samples. Because of extreme biosafety concerns and a level of discomfort handling a novel virus, DiaSorin EH&S did not initially approve of stocks of live virus or infected patient specimens in any company facilities. This presented challenges for completing kit development, considering the need for reliable reference material and material for a commercial kit positive control. Our initial solution for a kit positive control was to use synthetic gBlocks™ containing the assay target regions. This material had the advantages of being rapidly available and simple to order and obtain in large quantities. DiaSorin Molecular had previous experience with gBlocks and had confidence that they could be reproducibly manufactured, were stable and were compatible with the Simplexa™ Direct chemistry.

On February 21, 2020, contacts at NIH made us aware that the World Reference Center for Emerging Viruses and Arboviruses, Department of Microbiology and Immunology, University of Texas Medical Branch (UTMB) was culturing the virus isolated from the first U.S. patient in the state of Washington (USA_WA1/2020) and had small amounts of purified genomic RNA available. The material was requested through UTMB and was received by DiaSorin Molecular’s U.S. R&D in Cypress, California, on February 27. Simultaneously, the Italian R&D team gained access to purified viral genomic RNA from a positive patient sample on February 26. These sources of authentic positive control material that could be handled safely were desperately needed to complete development and evaluate assay performance. However, using this material presented a unique challenge for the Simplexa™ chemistry because naked viral RNA is not the typical assay specimen type for our direct assay format. The solution was to conduct limit of detection testing using the viral RNA in Universal Transport Medium (UTM) with the addition of RNasin^®^ RNase inhibitor to protect the viral RNA during the assay processing steps.

## Clinical Performance Challenges

With a working prototype Simplexa™ COVID-19 Direct assay, the next step was to test clinical specimens and find an appropriate comparator assay to evaluate clinical performance. An unknown at this stage was the sensitivity required for a SARS-CoV-2 assay. The CDC’s 2019 Coronavirus Diagnostic Panel was available. However, the initial lots were flawed and supplies were limited. Further, it was difficult to find labs that had the resources to test the CDC assay versus the DiaSorin Molecular Simplexa™ assay as all reagents available in hospital laboratories were being used to meet the growing testing demand. We chose to develop a panel of contrived positive samples using negative nasopharyngeal swab matrix spiked with extracted SARS-CoV-2 RNA plus RNasin^®^ as a substitute for positive clinical specimens to demonstrate assay performance. However, we still needed access to genuine patient samples to determine if the assay would be successful for clinical laboratory use.

## Crisis in Northern Italy

No one could have predicted that the second global hotspot after Wuhan would be the Lombardy region of Northern Italy, where DiaSorin S.p.A. corporate headquarters is located. The first Italian cases were reported around the third week of February 2020, and cases multiplied exponentially. Our assay development needed to be accelerated even more to address the growing urgent situation in Italy. We identified a few partner laboratories in Rome and Pavia, Italy, who were willing to assist and had validated the WHO Berlin protocol by Corman et al., published January 17, 2020. The DiaSorin Molecular Italian R&D team risked their health and exposure to COVID-19 patients that were starting to crowd the hospitals and placed LIAISON^®^ MDX instruments into the hospital labs to have access to fresh, positive patient specimens that would also have corresponding results with an established WHO comparator assay for performance benchmarking. This step allowed finalization of assay parameters so that by March 8, 2020, Italian R&D was successfully running the prototype assay with patient clinical specimens. These results enabled the clinical agreement studies required for initial CE marking of the assay in Italy and the European Union as well as the FDA Emergency Use Authorization.

The emergency continued to grow in Italy, and the entire country was placed on lockdown on March 9, 2020. Despite the setback, DiaSorin Molecular kept working diligently as the pandemic was taking hold, leaving families at home and risking falling ill. R&D employees were required to carry special permission to leave their homes to drive on the highway or cross the city of Milan to get to the R&D laboratory. We literally worked around the clock and around the globe, because as the Italian R&D team that designed the assay was racing to finalize the assay parameters and obtain clinical specimens, a second R&D team in California was simultaneously performing verification testing, and the manufacturing team in California was setting up kit production. Because of the lockdown and travel ban, we had to come up with creative ways to work together to take advantage of the 9-hour time difference between our locations. The Italian R&D team passed the baton to the California R&D and production teams at the end of the Italian day. The Italian team would begin the next day with results of the day of work in California. On March 11, 2020, the World Health Organization declared COVID-19 a pandemic due to cases in over 110 countries and territories worldwide. The first 12,000 Research Use Only (RUO) labeled tests were delivered to Italy the same week. The following week, DiaSorin Molecular had completed the activities required for CE mark self-declaration on March 20, 2020.

## Emergency Use Authorization & U.S. Government Support

Concurrently with development, DiaSorin Molecular’s pre-EUA submission questions were sent to the FDA on February 27, 2020. We communicated with FDA on an almost daily basis, working through challenges to establish the assay performance to the satisfaction of the FDA. DiaSorin Molecular submitted the Simplexa™ COVID-19 Direct kit EUA on Tuesday, March 17, 2020, at 6:08 PM Pacific time – a little less than two months from the first primer and probe orders. On Thursday, March 19, 2020, Simplexa™ COVID-19 Direct was granted Emergency Use Authorization by FDA. The assay was the fourth commercially available *in vitro* diagnostic (IVD) EUA kit and the first IVD shipments were sent to U.S. customers on Saturday, March 21, 2020. The assay performance has compared favorably against other commercially available FDA EUA and lab developed assays including those that use silica-based extraction (Cradic et al., 2020; Fung et al., 2020; Lieberman et al., 2020; Rhoads et al., 2020; Zhen et al., 2020).

With emerging infectious disease and pandemic preparedness, there is always a business risk. To help take on some of this risk, the U.S. Department of Health and Human Services’ Biomedical Advanced Research and Development Authority (BARDA) supports development of medical countermeasures such as diagnostic tests, antivirals, and vaccines. BARDA offered a fast-funding pathway at the start of the pandemic named the DRIVe EZ-BAA program. DiaSorin Molecular submitted an abstract requesting funding to complete the verification and validation of the Simplexa™ COVID-19 Direct assay using this program late on the evening of February 28, 2020. In record time, on March 11, DiaSorin Molecular was awarded a $697K BARDA development contract.

## Business Growth and Allocations

As COVID-19 cases were growing in early March 2020, there were only 12 LIAISON^®^ MDX system placements in Italy and 483 LIAISON^®^ MDX placements in the U.S., primarily in hospitals and reference laboratories. Our Cypress, California Simplexa™ kit manufacturing capacity was only 14,400 tests per day from a single manufacturing shift. In contrast, by May 2021, manufacturing had expanded to three shifts, building kits seven days per week for an output of over one million tests per month. LIAISON^®^ MDX placements had grown to 128 in Italy and 897 in the U.S.

Increasing production required hiring more employees in all manufacturing departments, from formulation to Quality Control testing to shipping. While the California manufacturing team was present in the facility in full force seven days a week, other departments worked from home starting from March 19, balancing the statewide stay at home order declared by California Governor Gavin Newsom with the fact that DiaSorin Molecular was an essential business in the pandemic fight. The facility was separated into three zones with separate entrances to isolate and protect kit production from other staff, with daily required symptom and temperature checks. There was a desire to test all employees with our Simplexa™ COVID-19 Direct test, but there was no guidance provided by the State of California’s Cal/OSHA even though this was necessary to protect the health of the production staff and their ability to continue kit production for the labs using our assays and Direct Amplification Discs. This challenge was solved by partnering with a testing service provider in San Diego, CA, to provide a blanket employee testing order from their lead physician, as well as nurses for weekly specimen collection, and by partnering with a CLIA-certified lab located close to DiaSorin Molecular to perform Simplexa™ COVID-19 Direct testing of our employees.

Careful allocation of kits to customers was managed daily starting from the EUA and CE marked launches of Simplexa™ COVID-19 Direct in March 2020. We were transparent with clients regarding weekly kit shipments and only took on new clients as kit manufacturing output increased. Direct Amplification Disc (DAD) production was ramped up in tandem with kit production. Fortunately for DiaSorin Molecular, the supply chain impacts experienced by other suppliers did not affect Simplexa™ COVID-19 Direct as no extraction reagents or plastic cartridges were required for producing the kit.

## Challenges After Launch

Typically, there are sources of inactivated viral reference materials and specimens with accompanying IVD test results to support lab validation. Since SARS-CoV-2 was a novel virus, external controls were not readily available for laboratories to validate EUA assay performance. With concerns about this novel agent and the limited supply of inactivated virus, many commercial suppliers turned to safer synthetic alternatives. DiaSorin Molecular proactively worked with Exact Diagnostics on a synthetic verification panel for laboratories to use for implementing our assay. As a result, external reference verification panel material was available immediately after launch to use for verifying assay performance.

Because of the massive increase in nasopharyngeal and nasal swab testing, transport media and collection swab shortages occurred in the U.S. and globally by April 2020. Desperate to have specimen collection media, labs were forced to resort to alternative transport media or manufacture their own based on shared recipes from the CDC (Centers for Disease Control and Prevention, 2020). Labs also validated readily available plastic cotton-tipped swabs with SARS-CoV-2 RT-PCR assays instead of plastic synthetic-tipped swabs that were considered the gold standard (Freire-Paspuel et al., 2020). Commercial assay manufacturers typically validate different transport media types with their assays because transport media contain multiple components that can be present at different concentrations that can impact downstream molecular diagnostic assay performance. A benefit of the Simplexa™ Direct assay chemistry is that it does not require a separate nucleic acid extraction step. Technically this has the advantage of no loss of specimen due to extraction efficiency impact and no extraction reagent supply expense or issues. However, caution must be taken with different transport media types because the Simplexa™ chemistry comes into direct contact with the transport medium. Differences in salt, pH or media components such as those that denature proteins can impact Simplexa™ performance. Without proper validation, some of those alternative media types did, in fact, impact the Limit of Detection leading to reduced test sensitivity. Others that contained viral deactivation components also completely inactivated the reverse transcriptase and Taq polymerase enzymes that are components of the Simplexa™ Direct chemistry active ingredients. DiaSorin Molecular formally validated 0.9% saline as a collection media alternative to allow labs a consistent and reliable option to use when the other on label transport media were in short supply.

## Supply Chain Challenges From a Manufacturing Perspective

Within 30 days of EUA kit launch in March 2020, Simplexa™ COVID-19 Direct reagent production increased by 141%, and production ramp-up was started for the DAD consumable required to perform the test. By January 2021, during peak demand period, production increased 2999% for DAD consumables and 433% for COVID-19 reagents. All in all, during the COVID-19 pandemic, DiaSorin Molecular packaged and shipped more than ten times as many kits per month compared to the highest prior production month in previous history. Remarkably, support was also maintained for the other >100 non-COVID-19 products and there were no customer backorders during this time.

Stepping up to the pandemic production demand required expansion of the supply chain base, increased production throughput, additional equipment sourcing, expansion of production areas, and headcount increases, all of which were performed simultaneously. The Supply Chain organization was transformed during 2020 to secure raw materials at a time where the global supply chain suffered severe interruptions, depletion, and heightened demand. To ensure business continuity, DiaSorin Molecular developed relationships with multiple backup suppliers for dual sourcing of all critical supplies; this effort yielded 25 new key suppliers, which were used to alleviate constraints for commodities, including plastics, pipettes, tank liners, oligonucleotides, resin, dry ice, and more. To combat supplier constraints at the contract manufacturer for discs, production was partially insourced and a new supply chain was developed to match the contract manufacturers’ maximum volume in consumable production. In five months, a second DAD production line was implemented with new automation for manufacturing and inspection of the DADs, and 12 new pieces of equipment were installed and validated. With demand increasing, this was followed by implementing a third manufacturing line with automation, expanding the cleanroom to make space for this third line, and installing and validating 18 new pieces of equipment. A new DAD mold was also validated and implemented in January 2021, with a new mold maker vendor to strengthen our supply chain. Upgrades were also made to the warehouse to increase storage capacity for raw materials and finished goods. The company invested in expanded frozen storage capacity to provide critical backup capabilities to preserve finished goods. Manufacturing Engineering and Production teams further scaled-up kit production by fine-tuning the speed and accuracy of automated dispensing equipment to minimize reagent waste and increase throughput. The Technical Operations team performed process validations to substantially increase lot sizes. These expansions required increased staff, and Production personnel increased by 150% starting from a single shift, Monday through Friday in March 2020 to three shifts, seven days a week during peak production.

## Future Perspectives and Recommendations

Understanding the challenges faced by DiaSorin Molecular to bring a sensitive and specific SARS-CoV-2 RT-PCR assay to market as quickly as possible can help us prepare for future pandemics. We can reflect on the supply chain constraints that were faced internally, and by our customers. Rapid assay development was enabled by having a core Simplexa™ Direct chemistry in which to drop in primer and probe designs for a novel pathogen. The public sharing and access to databases with the virus sequence also allowed for the fastest possible target design for a molecular assay. The willingness of hospital labs to closely work with a commercial partner for access to precious clinical specimens was paramount. The availability of the FDA for discussions regarding the regulatory path and the support received from BARDA was also crucial. Pressure to manufacture as many kits as possible allowed us to quickly expand and add on new manufacturing lines and creatively overcome supply challenges. One aspect that remains critical is the worldwide shortage of the resin used for plastics manufacturing. Because disposable, medical-grade plastics are used throughout the healthcare industry, this is an area that needs attention in order to be prepared for surges of SARS-CoV-2 and pandemics in the future. Another consideration for future global pandemic preparedness is the availability of reference standards and mandatory rapid performance assessments to guarantee the quality of the supplies on the market for every country in the world (Freire-Paspuel and Garcia-Bereguiain, 2021). The successful rapid assay launch, production scale-up, and continuity of supply to our customers during the COVID-19 pandemic response is a testament to the DiaSorin Molecular team’s capabilities, skillset, and determination across all groups within DiaSorin that supported their efforts. It would not have been possible without the courage and cooperation of the outstanding and resilient teams in Italy and the U.S.

## Data Availability Statement

The original contributions presented in the study are included in the article. Further inquiries can be directed to the corresponding author.

## Author Contributions

MT was responsible for writing a majority of the article. GM was responsible for writing about DiaSorin Italy experience. VA was responsible for writing about DiaSorin Molecular manufacturing experience. All authors contributed to the article and approved the submitted version.

## Funding

This project has been funded in whole or in part with Federal funds from the Department of Health and Human Services; Office of the Assistant Secretary for Preparedness and Response; Biomedical Advanced Research and Development Authority, under Contract No. 75A5012OC00017.

## Conflict of Interest

MT and VA are all employees of DiaSorin Molecular LLC. GM is an employee of DiaSorin S.p.A.

## Publisher’s Note

All claims expressed in this article are solely those of the authors and do not necessarily represent those of their affiliated organizations, or those of the publisher, the editors and the reviewers. Any product that may be evaluated in this article, or claim that may be made by its manufacturer, is not guaranteed or endorsed by the publisher.
